# Assessment of universal health coverage for adults aged 50 years or older with chronic illness in six middle-income countries

**DOI:** 10.2471/BLT.15.163832

**Published:** 2016-03-03

**Authors:** Christine Goeppel, Patricia Frenz, Linus Grabenhenrich, Thomas Keil, Peter Tinnemann

**Affiliations:** a Global Health Science Unit, Institute of Social Medicine, Epidemiology and Health Economics, Charité Universitätsmedizin Berlin, Luisenstraße 57, Berlin, 10117, Germany.; b Escuela de Salud Pública, Universidad de Chile, Santiago de Chile, Chile.

## Abstract

**Objective:**

To assess universal health coverage for adults aged 50 years or older with chronic illness in China, Ghana, India, Mexico, the Russian Federation and South Africa.

**Methods:**

We obtained data on 16 631 participants aged 50 years or older who had at least one diagnosed chronic condition from the World Health Organization Study on Global Ageing and Adult Health. Access to basic chronic care and financial hardship were assessed and the influence of health insurance and rural or urban residence was determined by logistic regression analysis.

**Findings:**

The weighted proportion of participants with access to basic chronic care ranged from 20.6% in Mexico to 47.6% in South Africa. Access rates were unequally distributed and disadvantaged poor people, except in South Africa where primary health care is free to all. Rural residence did not affect access. The proportion with catastrophic out-of-pocket expenditure for the last outpatient visit ranged from 14.5% in China to 54.8% in Ghana. Financial hardship was more common among the poor in most countries but affected all income groups. Health insurance generally increased access to care but gave insufficient protection against financial hardship.

**Conclusion:**

No country provided access to basic chronic care for more than half of the participants with chronic illness. The poor were less likely to receive care and more likely to face financial hardship in most countries. However, inequity of access was not fully determined by the level of economic development or insurance coverage. Future health reforms should aim to improve service quality and increase democratic oversight of health care.

## Résumé

**Objectif:**

Évaluer la couverture sanitaire universelle pour des adultes de 50 ans ou plus, atteints de maladie chronique, en Afrique du Sud, en Chine, dans la Fédération de Russie, au Ghana, en Inde et au Mexique.

**Méthodes:**

À partir de l'étude de l’OMS sur le vieillissement et la santé des adultes dans le monde (SAGE), nous avons obtenu des données sur 16 631 participants, âgés de 50 ans ou plus, chez qui au moins une maladie chronique a été diagnostiquée. L'accès à des soins chroniques de base et les difficultés financières ont été évalués, et des analyses par régression logistique ont permis de déterminer l'influence d’avoir une assurance maladie et l'influence de vivre en milieu rural ou urbain.

**Résultats:**

La part pondérée des participants bénéficiant d'un accès à des soins chroniques de base varie entre 20,6% au Mexique et 47,6% en Afrique du Sud. Les taux d'accès sont inégalement répartis et en défaveur des personnes pauvres, sauf en Afrique du Sud, où les soins de santé primaires sont gratuits pour tous. Le fait d'habiter en milieu rural n'a pas d'incidence sur cet accès. La proportion des participants ayant dû assumer des dépenses directes catastrophiques pour leur dernière consultation ambulatoire s'établit entre 14,5% en Chine et 54,8% au Ghana. Dans la plupart de ces pays, les difficultés financières sont plus fréquentes chez les personnes pauvres, mais elles affectent toutes les tranches de revenus. Les assurances maladie améliorent généralement l'accès aux soins mais offrent une protection insuffisante contre les difficultés financières.

**Conclusion:**

Aucun des pays étudiés ne permet à plus de la moitié des participants atteints de maladie chronique d’accéder aux soins chroniques de base. Dans la majorité de ces pays, les personnes pauvres sont moins susceptibles de bénéficier de soins et plus exposées aux difficultés financières. Néanmoins, l'inégalité en termes d'accès aux soins n'est pas entièrement déterminée par le niveau de développement économique ou par la couverture d'assurance. Les réformes à venir des systèmes de santé devraient viser à améliorer la qualité des services et à favoriser une supervision plus démocratique des soins de santé.

## Resumen

**Objetivo:**

Evaluar la cobertura sanitaria universal para adultos de 50 años o más con enfermedades crónicas en China, Federación de Rusia, Ghana, India, México y Sudáfrica.

**Métodos:**

Se obtuvieron datos de 16 631 participantes de 50 años o más diagnosticados con, al menos, una enfermedad crónica del Estudio de la OMS sobre envejecimiento y salud de los adultos en el mundo. Se evaluaron el acceso a atención crónica básica y las dificultades económicas y se determinó la influencia del seguro sanitario y de la residencia rural o urbana mediante un análisis de regresión logística.

**Resultados:**

La proporción ponderada de participantes con acceso a atención crónica básica varió desde un 20,6% en México a un 47,6% en Sudáfrica. Las tasas de acceso estaban desigualmente distribuidas y la población pobre se encontraba en desventaja, salvo en Sudáfrica, donde la atención sanitaria primaria es gratuita para todo el mundo. La residencia rural no afectaba al acceso. La proporción de gastos catastróficos directos durante la última visita ambulatoria varió desde un 14,5% en China a un 54,8% en Ghana. Las dificultades económicas eran más comunes entre los pobres en la mayoría de países, pero afectaron todos los grupos de ingresos. Por norma general, los seguros sanitarios aumentaron el acceso a la asistencia, pero no ofrecieron la suficiente protección frente a las dificultades económicas.

**Conclusión:**

Ningún país ofrecía acceso a atención crónica básica a más de la mitad de los participantes con enfermedades crónicas. En la mayoría de los países, los pobres tenían menos posibilidades de recibir asistencia y más de sufrir dificultades económicas. No obstante, la desigualdad en el acceso no estaba totalmente condicionada por el nivel de desarrollo económico o la cobertura del seguro. Las futuras reformas sanitarias deberían tener como objetivo mejorar la calidad del servicio y aumentar la supervisión democrática de la atención sanitaria.

## ملخص

**الغرض:**

لتقييم مستوى التغطية الصحية الشاملة للبالغين بسن 50 عامًا أو أكبر والذين يعانون من أمراض مزمنة في الصين وغانا والهند والمكسيك والاتحاد الروسي وجنوب أفريقيا.

**الطريقة:**

حصلنا على بيانات خاصة بعدد 16,631 مشاركًا تبلغ أعمارهم 50 عامًا أو أكبر والمصابين بحالة مرضية مزمنة واحدة على الأقل تم تشخيصها في الدراسة التي أجرتها منظمة الصحة العالمية بشأن التقدم في السن على الصعيد العالمي وصحة البالغين. تم تقييم سبل الحصول على الرعاية الأساسية للأمراض المزمنة والصعوبات المالية وتم تحديد تأثير التأمين الصحي والإقامة في المناطق الريفية أو الحضرية من خلال تحليل التحوف اللوجيستي.

**النتائج:**

تراوحت نسبة الأرجحية للمشاركين الحاصلين على الرعاية الأساسية للأمراض المزمنة من ‏20.6% في المكسيك إلى ‏47.6% في جنوب أفريقيا. لم يتم توزيع معدلات الاستفادة من الرعاية الصحية الأساسية بصورة متكافئة ما أدى إلى حرمان الفقراء من الحصول عليها، وسجلت جنوب أفريقيا استثناءً لهذه الحالة حيث تُقدم فيها الرعاية الصحية الأساسية مجانًا للجميع. ولم تؤثر الإقامة في المناطق الريفية على سبل الاستفادة. وقد تراوحت نسبة الإنفاق الشخصي الباهظة للزيارة الأخيرة للمريض الذي يتلقى العلاج خارج المستشفى من 14.5‏% في الصين إلى 54.8‏% في غانا. كانت الصعوبات المالية هي الأكثر شيوعًا بين الفقراء في معظم البلدان ولكنها أثرت على جميع فئات الدخل. وأدى التأمين الصحي إلى زيادة سبل الحصول على الرعاية بشكل عام ولكنه لم يمنح الحماية الكافية في مواجهة الصعوبات المالية.

**الاستنتاج:**

لم توفر أي بلد سبلاً للحصول على الرعاية الأساسية للأمراض المزمنة لعدد يزيد عن نصف المشاركين الذين يعانون من أمراض مزمنة. كان الفقراء الأقل احتمالاً لتلقي الرعاية والأكثر احتمالاً لمواجهة صعوبات مالية في معظم البلدان. وعلى الرغم من ذلك، لم يتم تحديد حالات عدم الإنصاف في توفير سبل الحصول على الرعاية بالكامل من خلال مستوى التنمية الاقتصادية أو التغطية التأمينية. وينبغي على الإصلاحات في الخدمات الصحية في المستقبل أن تهدف إلى تطوير جودة الخدمات وزيادة الرقابة الديموقراطية على الرعاية الصحية.

## 摘要

**目的:**

旨在评估俄罗斯、加纳、墨西哥、南非、印度和中国 50 岁或以上慢性病患者全民医疗覆盖的情况。

**方法:**

我们从《世界卫生组织全球老龄化和成年人健康研究》中获取 16631 名 50 岁或以上且经诊断患有至少一种慢性病的参与者数据。 我们评估了获得基础慢性病治疗和资金的困难，并通过逻辑回归分析确定了城乡居民医疗保险的影响。

**结果:**

接受基础慢性病治疗的参与者所占加权比例范围从墨西哥的 20.6% 到南非的 47.6%。 除主要医疗护理全民免费的南非以外，其他国家接受率分布不均匀且贫困群体处于弱势地位。 农村居民并不影响接受率。 最后一次看门诊支出大量现金的比例范围从中国的 14.5% 到加纳的 54.8%。 在大多数国家内，资金困难在贫困群体中更为普遍，但所有收入群体都会受到影响。 医疗保险通常能提高护理接受率，但无法充分解决资金困难问题。

**结论:**

半数以上的慢性病参与者，未能从其国家获得基础慢性病治疗。 在大多数国家，贫困群体接受治疗的可能性更低且面临资金困难的可能性更高。 但是，接受率不均并不完全由经济发展水平或保险覆盖率决定。 未来医疗改革应该以提高服务质量和改进医疗护理的民主监督为目标。

## Резюме

**Цель:**

Оценить обеспечение всеобщего охвата услугами здравоохранения взрослого населения в возрасте от 50 лет, страдающего хроническими заболеваниями, в Гане, Индии, Китае, Мексике, Российской Федерации и Южной Африке.

**Методы:**

На основе исследования Всемирной организацией здравоохранения по проблемам глобального старения и здоровья взрослых людей были получены данные по 16 631 участнику исследования в возрасте от 50 лет, у которых диагностировано по меньшей мере одно хроническое заболевание. В результате проведения анализа с использованием модели логистической регрессии были оценены доступность базовой медицинской помощи для лечения хронических заболеваний и финансовые трудности, а также определено влияние наличия медицинской страховки и проживания в сельских или городских условиях.

**Результаты:**

Взвешенная доля участников, имеющих доступ к базовой медицинской помощи для лечения хронических заболеваний, варьировалась от 20,6% в Мексике до 47,6% в Южной Африке. Доступность помощи была неравномерно распределена; самая низкая степень доступности была зафиксирована для малоимущего населения, за исключением населения Южной Африки, где первичная медицинская помощь бесплатна для всех. Проживание в сельской местности не сказалось на доступности. Доля людей, превысивших предел расходов из собственных средств при последнем обращении в поликлинику, варьировалась от 14,5% в Китае до 54,8% в Гане. Финансовые трудности в большем числе случаев испытывало малоимущее население в большинстве стран, но они затронули все группы по уровню доходов. Наличие медицинской страховки в целом способствовало повышению доступности медико-санитарной помощи, однако страховка не предоставляла достаточной защиты от финансовых трудностей.

**Вывод:**

Ни в одной стране большей части участников, страдающих хроническими заболеваниями, не предоставлялся доступ к базовой медицинской помощи для лечения хронических заболеваний. В большинстве стран вероятность получения помощи малоимущими участниками была ниже, а вероятность столкновения с финансовыми трудностями — выше. Тем не менее неравномерность доступа не в полной мере определялась степенью экономического развития или наличия страховки. Будущие реформы здравоохранения должны ставить перед собой цель улучшения качества и повышения общедоступности медико-санитарной помощи.

## Introduction

The World Health Assembly in 2005 and the United Nations General Assembly in 2012 called for universal health coverage to reinforce the human right to health. All Member States were requested to guarantee affordable promotive, preventive, curative and rehabilitative health care of the highest attainable standard for everyone, without distinction.^1^^,^^2^ However, over the past three decades, market deregulation and political crises have led to increased inequalities in income and opportunity in many countries. These inequalities are reflected in highly fragmented health and social security systems, which are increasingly differentiated by socioeconomic strata, and in setbacks for publicly funded health services. As a result, poorer social groups, including the historically marginalized and those more recently excluded from social protection systems, are forced either to forego care or to pay for access to increasingly costly health-care services. Consequently, access to health care in many countries has deteriorated to such an extent that health problems have become a threat to social development and cohesion.^3^

At the same time, the rise in chronic noncommunicable diseases makes international development goals more difficult to achieve and complicates strategies for attaining universal health coverage. Moreover, the epidemiological transition, which is characterized by a sharp increase in population growth and a change in the leading causes of death, is rapidly accelerating in the poorest strata of society, where people are less likely to have access to appropriate services and are at risk of catastrophic health-care costs.^4^^,^^5^ Many middle-income countries, including those we investigated, have scaled up efforts to achieve universal health coverage through substantial health reforms with a particular emphasis on the poor and vulnerable. These reforms have adopted a range of voluntary and social health insurance schemes in their attempt to increase service utilization while avoiding financial hardship and encouraging equity of access.^6^

The aim of this study was to investigate gaps in universal health coverage for specific socioeconomic groups by focusing on older adults with chronic illness in China, Ghana, India, Mexico, the Russian Federation and South Africa. We examined five key issues: (i) access to basic chronic care; (ii) protection against financial hardship; (iii) the influence of health insurance schemes; (iv) the influence of place of residence; and (v) general satisfaction with the health-care system. We also examined progress towards universal health coverage in the six countries.

## Methods

The World Health Organization’s (WHO’s) Study on Global Ageing and Adult Health (SAGE) provides comparable, publicly available data on adults aged 50 years and older based on nationally representative household surveys for six countries: China, Ghana, India, Mexico, the Russian Federation and South Africa.^7^ These countries have some of the fastest growing economies globally and together contain more than 40% of the world’s population spread over four regions.^8^ We analysed cross-sectional data from wave 1 of the study carried out between 2007 and 2010. The response rate in individual surveys ranged from 52% in Mexico to 93% in China.^7^

Our study population consisted of all participants in the Study on Global Ageing and Adult Health who could be categorized as older adults with chronic illness: they were aged 50 years and older and reported being diagnosed with at least one chronic disease, such as arthritis, hypertension, stroke, angina, diabetes, chronic lung disease, asthma or depression. Universal health coverage was assessed on three dimensions: (i) access to basic chronic care; (ii) income-related equity of access; and (iii) protection against financial hardship. We also investigated the influence of health insurance on both access to basic chronic care and financial hardship and the influence of rural or urban habitation on access and we compared levels of satisfaction with the health system between people who did and did not use outpatient care.

Access to basic chronic care was assessed using a compound indicator with three components: (i) the provision of treatment, such as medications or advice on physical activity or diet, for each of the patient’s conditions; (ii) visiting outpatient services for the chronic condition or conditions one or more times in the last reported year; and (iii) maintenance of a stable health state after the last outpatient visit. In the surveys, health-care providers were categorized as medical doctors, nurses, physiotherapists or traditional practitioners. Equity of access was assessed on the basis of equal treatment for equal health needs.^9^ Since all study participants had chronic conditions, they all needed access to health care. Therefore, any income-related disparity in access to basic chronic care within a country indicated the existence of an inequity.

Financial hardship was defined in two ways: (i) catastrophic household spending on health in the last reported year of more than 30% of annual average household income, after the deduction of food expenditure – health expenditure included prepayments and out-of-pocket expenses; and (ii) catastrophic out-of-pocket expenditure for the last outpatient visit of more than 30% of annual household per capita income, after the deduction of food expenditure – expenditure on the outpatient visit included doctor fees and the cost of medications, diagnosis and transport. For our analysis, we based household income quintiles on annual household per capita income.^10^ A person with any type of health insurance was classified as insured. Dissatisfaction with the health-care system was assessed using two indicators: (i) dissatisfaction with health-care services; and (ii) insufficient involvement in health-care decision-making.

### Statistical analysis

We adjusted study data for differences between countries in survey design and data collection in Stata version 13.1 (StataCorp. LP, College Station, United States of America) using person-level analysis weights based on selection probabilities in the survey sampling design and a post-stratification factor. All percentage estimates are weighted. Differences between countries in access to basic chronic care and financial hardship were described using weighted population means. Within countries, we assessed the effect of poverty by stratifying data by household income quintile and looked particularly at differences between the poorest quintile and the population mean. Differences in equity of access between countries were compared using concentration curves and indices. We employed logistic regression modelling to estimate: (i) the effect of health insurance on access to basic chronic care and financial hardship; (ii) the effect of rural or urban residence on access; and (iii) the effect of using outpatient services on dissatisfaction with the health system. Models were adjusted for sex, age, place of residence, educational level, income quintile, comorbidity and insurance. Finally, we examined the effect of macroeconomic and social factors on universal health coverage in different countries by determining whether gross national income per capita, public health expenditure per capita or the Gini coefficient was associated with access to basic chronic care without incurring catastrophic out-of-pocket expenditure for the last outpatient visit.^11^^–^^17^

## Results

Our study population comprised 16 631 individuals who formed nationally representative samples. The proportion of females was highest in South Africa (62.8%; 95% confidence interval, CI: 59.2 to 66.3) and lowest in India (47.5%; 95% CI: 44.6 to 50.4) and the mean age of participants ranged from 62.3 years (standard difference, SD: 0.3) in India to 66.3 years (SD: 0.4) in Ghana (Table 1). The proportion living in an urban area ranged from 33.5% (95% CI: 26.8 to 41.0) in India to 81.6% (95% CI: 74.5 to 87.1) in Mexico and the proportion with health insurance ranged from 99.7% (95% CI: 99.4 to 99.9) of Russians to only 5.2% (95% CI: 3.6 to 7.3) of Indians.

**Table 1:** Demographic and socioeconomic characteristics of participants, study of universal health coverage for adults aged 50 years or older with chronic illness, 2007–2010

Characteristic	China	Ghana	India	Mexico	Russian Federation	South Africa
**Study population,^a^*n***	6558	1327	2623	1341	2916	1866
**Prevalence of chronic disease in the SAGE population,^b^ % (95% CI)**	50.5 (48.5 to 52.4)	32.0 (29.8 to 34.4)	41.8 (39.2 to 44.4)	55.0 (49.5 to 60.3)	72.7 (69.1 to 76.0)	50.5 (47.3 to 53.7)
**Age in years, mean (SE)**	64.2 (0.2)	66.3 (0.4)	62.3 (0.3)	64.8 (0.9)	65.2 (0.7)	62.4 (0.4)
**Female sex, % (95% CI)**	54.5 (53.2 to 55.7)	54.6 (50.9 to 58.2)	47.5 (44.6 to 50.4)	62.0 (54.3 to 69.1)	61.9 (57.7 to 66.0)	62.8 (59.2 to 66.3)
**Comorbidity,^c^ % (95% CI)**	38.4 (36.6 to 40.3)	29.4 (26.4 to 32.6)	37.5 (34.5 to 40.7)	36.1 (29.0 to 44.0)	63.3 (59.0 to 67.4)	42.7 (38.6 to 46.9)
**Urban residence, % (95% CI)**	55.0 (53.1 to 56.9)	52.3 (48.4 to 56.3)	33.5 (26.8 to 41.0)	81.6 (74.5 to 87.1)	71.3 (58.6 to 81.3)	68.7 (63.7 to 73.3)
**Health insurance, % (95% CI)**						
None	10.9 (9.2 to 12.8)	52.5 (48.7 to 56.4)	94.9 (92.7 to 96.4)	27.4 (17.1 to 40.8)	0.2 (0.1 to 0.6)	79.8 (76.4 to 82.8)
Mandatory	76.5 (73.1 to 79.5)	2.3 (1.4 to 3.8)	2.3 (1.4 to 3.6)	53.8 (43.1 to 64.1)	98.3 (96.4 to 99.2)	7.0 (5.2 to 9.4)
Voluntary	5.9 (4.8 to 7.3)	43.2 (39.3 to 47.1)	2.7 (1.5 to 4.7)	18.6 (13.0 to 25.9)	0.7 (0.2 to 2.3)	9.9 (7.4 to 13.0)
Both mandatory and voluntary	6.8 (4.6 to 9.8)	2.0 (1.2 to 3.2)	0.2 (0.1 to 0.5)	0.3 (0.1 to 1.2)	0.7 (0.2 to 2.8)	3.3 (2.1 to 5.1)
**Educational level, % (95% CI)**						
Less than primary school	38.2 (35.2 to 41.3)	55.4 (51.3 to 59.4)	43.8 (39.6 to 48.1)	53.0 (44.8 to 61.1)	1.8 (1.1 to 2.9)	44.9 (40.3 to 49.5)
Primary school	21.0 (18.4 to 23.9)	10.0 (8.0 to 12.4)	17.9 (14.8 to 21.4)	23.5 (17.6 to 30.8)	6.4 (4.1 to 9.9)	24.6 (21.2 to 28.5)
Secondary school	21.3 (19.5 to 23.2)	6.5 (5.0 to 8.6)	16.6 (13.8 to 19.8)	15.2 (8.6 to 25.5)	19.9 (15.1 to 25.8)	16.4 (13.3 to 20.1)
More than secondary school	19.5 (16.5 to 22.8)	28.1 (25.0 to 31.5)	21.7 (17.6 to 26.4)	8.2 (4.8 to 13.6)	71.8 (64.7 to 78.0)	14.1 (10.9 to 18.1)

CI: confidence interval; SAGE: Study on Global Ageing and Adult Health; SE: standard error.

^a^ The study population comprised chronically ill participants in wave 1 of the World Health Organization’s SAGE.

^b^ Percentage of participants in wave 1 of SAGE, which included only people aged 50 years or older, who were diagnosed with a chronic condition.

^c^ Comorbidity was defined as having at least two chronic conditions.

Notes: Chronic illness was defined as being diagnosed with at least one chronic disease. Percentages are weighted.

**Fig. 1:**
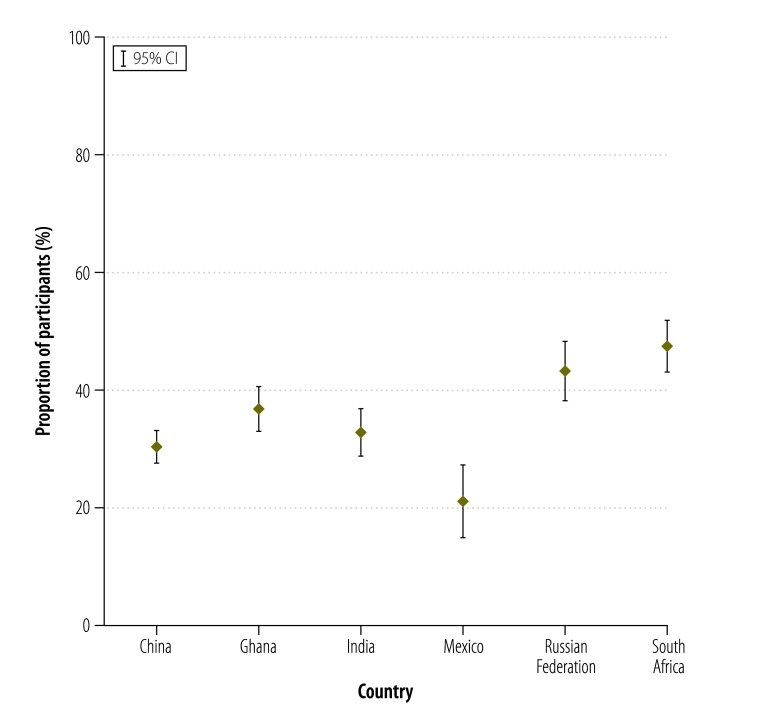
Access to basic chronic care by adults aged 50 years or older with chronic illness in six middle-income countries, 2007–2010

**Fig. 2:**
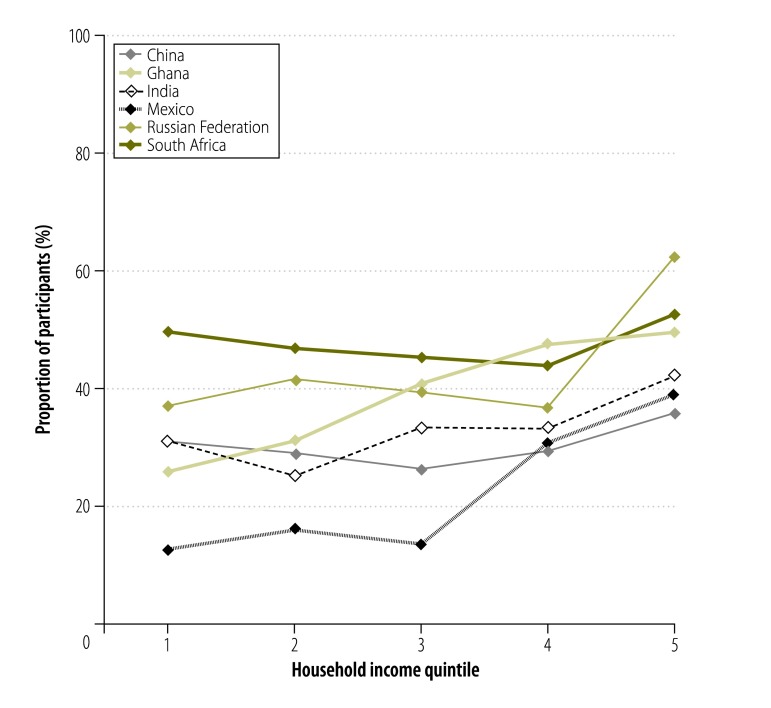
Access to basic chronic care by adults aged 50 years or older with chronic illness, by household income quintile, in six middle-income countries, 2007–2010

Notes: Chronic illness was defined as being diagnosed with at least one chronic disease. Household income quintiles were based on annual household per capita income. Quintile 1 represents the poorest and quintile 5 represents the richest.

**Fig. 3:**
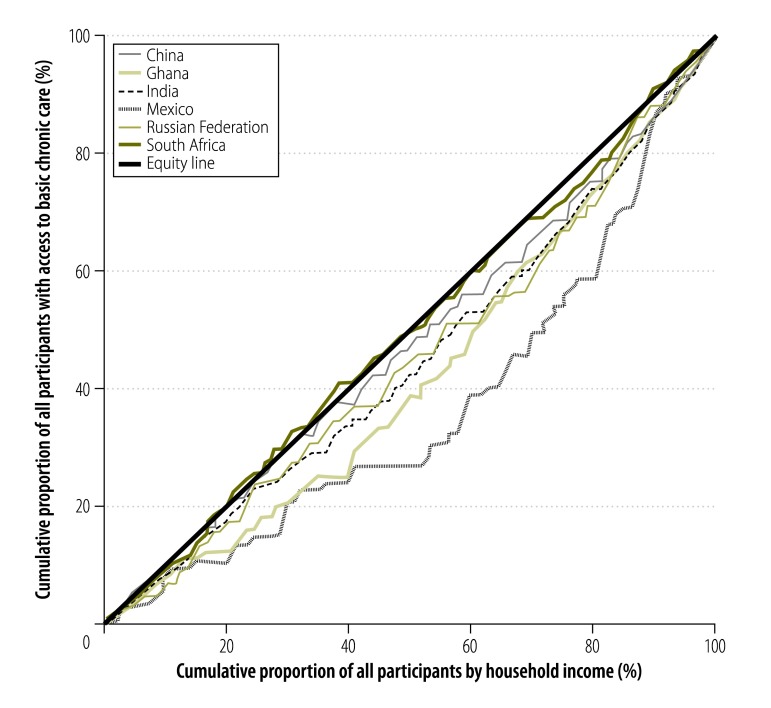
Concentration curves for access to basic chronic care, by household income and country, in six middle-income countries, 2007–2010

Notes: A curve lying below the equity line indicates that the poor have less access to basic chronic care than the rich: the greater the deviation from the equity line, the greater the inequity. Chronic illness was defined as being diagnosed with at least one chronic disease.

**Fig. 4:**
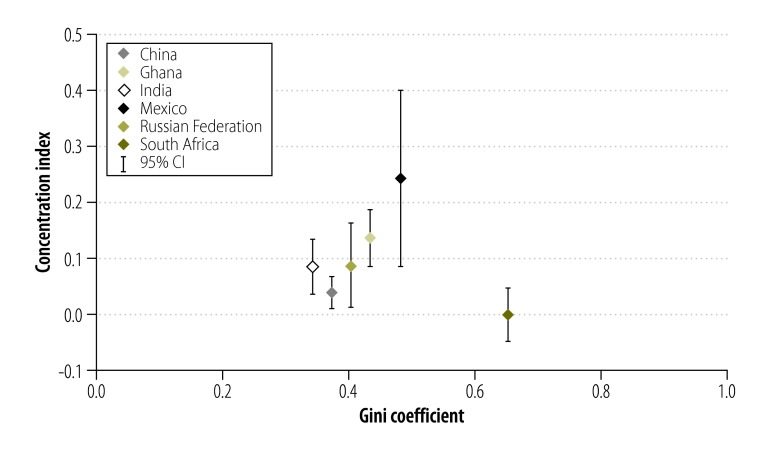
Concentration index for access to basic chronic care for adults aged 50 years or older with chronic illness, by Gini coefficient, in six middle-income countries, 2007–2010

Access to basic chronic care varied widely: the proportion of participants with access to basic chronic care was highest in South Africa (47.6%; 95% CI: 43.3 to 51.9) and the Russian Federation (43.5%; 95% CI: 38.6 to 48.4). The figures for Ghana, India and China were 36.9% (95% CI: 33.3 to 40.6), 32.9% (95% CI: 29.2 to 36.8) and 30.5% (95% CI: 27.8 to 33.4), respectively, and in Mexico it was only 20.6% (95% CI: 15.1 to 27.4; Fig. 1). Access rates were highest for the richest household income quintile in all countries. In Ghana, there was a continuous gradient from poor to rich. In India, the access rate decreased from the poorest to the second poorest quintile and then increased continuously to the richest quintile. There was a sharp increase in the access rate for the fourth and fifth income quintiles in Mexico and, for the richest quintile, in the Russian Federation. In China and South Africa, only small changes in the proportion with access were observed across the quintiles (Fig. 2). Moreover, the concentration curves for all countries except South Africa lay below the equity line and tested dominant (Fig. 3, available at: http://www.who.int/bulletin/volumes/94/4/15-163832), which indicates that the rich had disproportionate access to chronic care. Inequity of access was most pronounced for Mexico. The related concentration index varied substantially from 0.003 (95% CI: −0.045 to 0.050) for South Africa to 0.249 (95% CI: 0.087 to 0.403) for Mexico – higher values indicate greater inequity between rich and poor (Fig. 4).

CI: confidence interval.

Note: Chronic illness was defined as being diagnosed with at least one chronic disease.

CI: confidence interval.

Notes: Chronic illness was defined as being diagnosed with at least one chronic disease. The concentration index varies from –1 to +1 and is 0 for equity of access to basic chronic care. A value greater than 0 indicates that the rich have better access to care than the poor. The Gini coefficient measures the extent to which the distribution of income or consumption expenditure among individuals or households within an economy deviates from a perfectly equal distribution: a coefficient of 0 represents perfect equality, whereas a coefficient of 1 implies perfect inequality.

**Fig. 5:**
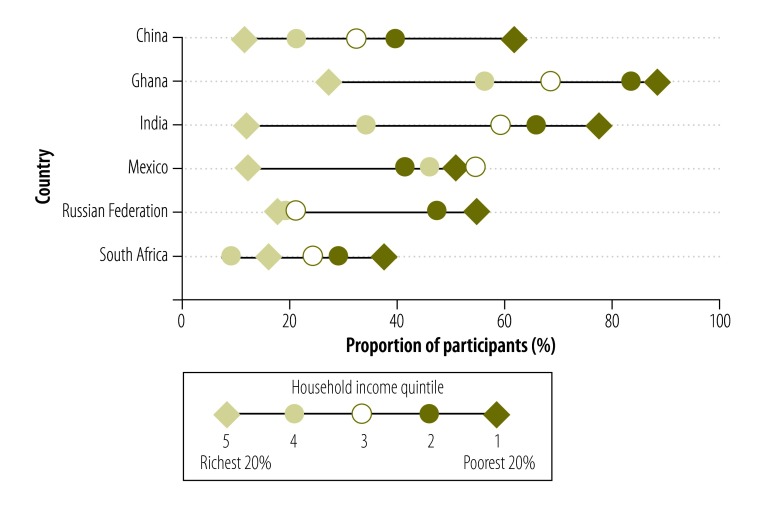
Catastrophic health spending in last reported year by adults aged 50 years or older with chronic illness, by household income and country, in six middle-income countries, 2007–2010

Notes: Chronic illness was defined as being diagnosed with at least one chronic disease. Catastrophic health spending in the last year was defined as the household spending more on health in the last reported year than 30% of annual household income, after deduction of food expenditure. Household income quintiles were based on annual household per capita income. The greater the distance between quintiles, the greater the effect of household income on the likelihood of catastrophic health spending.

**Fig. 6:**
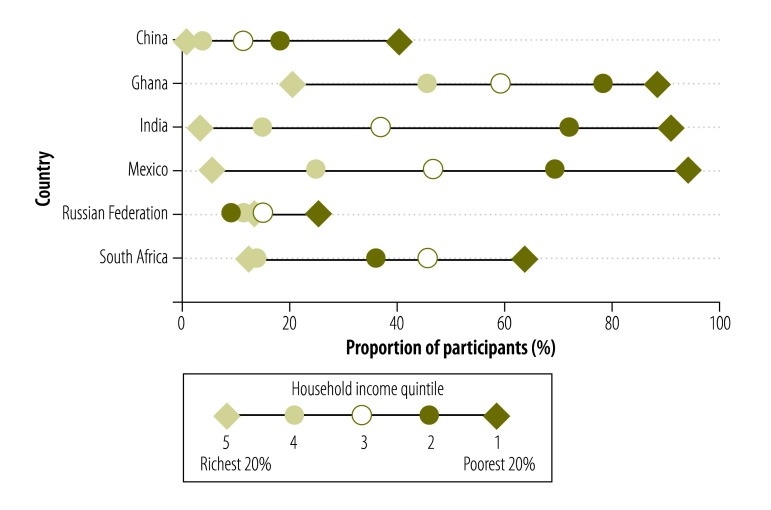
Catastrophic out-of-pocket expenditure for last outpatient visit for adults aged 50 years or older with chronic illness, by household income and country, in six middle-income countries, 2007–2010

Notes: Chronic illness was defined as being diagnosed with at least one chronic disease. Catastrophic out-of-pocket expenditure was defined as expenditure more than 30% of annual household per capita income, after the deduction of food expenditure at the last outpatient visit. Household income quintiles were based on annual household per capita income. The greater the distance between quintiles, the greater the effect of household income on the likelihood of catastrophic out-of-pocket expenditure.

The proportion of households that faced catastrophic health spending in the last reported year varied between 23.5% (95% CI: 19.3 to 28.3) in South Africa and 65.5% (95% CI: 60.6–69.8) in Ghana. Financial hardship was present in all socioeconomic strata, though the proportion affected was generally highest in the poorest household income quintile, except in Mexico, where the proportion affected was highest in the third quintile (Fig. 5, available at: http://www.who.int/bulletin/volumes/94/4/15-163832). The proportion with catastrophic out-of-pocket expenditure for the last outpatient visit varied between 14.5% (95% CI: 12.7−16.4) in China and 54.8% (95% CI: 49.1 to 60.4) in Ghana. The proportion of the poorest quintile that experienced such expenditure ranged from 25.5% (95% CI: 10.1 to 51.0) in the Russian Federation to 94.5% (95% CI: 81.6 to 98.5) in Mexico (Fig. 6, available at: http://www.who.int/bulletin/volumes/94/4/15-163832).

Participants with health insurance were significantly more likely to have access to basic chronic care than those without in China, Ghana, India and Mexico but not South Africa (Table 2). In India, insurance increased the odds more than threefold. Nevertheless, insurance did not necessarily protect against financial hardship. In China, Ghana, India and South Africa, the risk of catastrophic health spending in the last reported year was the same or even higher for the insured as the uninsured. In India, health insurance was also associated with a higher risk of catastrophic out-of-pocket expenditure for the last outpatient visit. However, in Mexico insurance coverage was associated with a significantly lower risk of catastrophic out-of-pocket expenditure for the last outpatient visit (odds ratio, OR: 0.35; 95% CI: 0.14 to 0.84) and a nonsignificantly lower risk of catastrophic health spending in the last reported year (OR: 0.49; 95% CI: 0.22 to 1.07). In Ghana, the insured had a significantly lower risk of catastrophic out-of-pocket expenditure (OR: 0.38; 95% CI: 0.23 to 0.62) but a nonsignificantly higher risk of catastrophic health spending in the last year (OR: 1.22; 95% CI: 0.86 to 1.73). Living in a rural rather than an urban area was not associated with a lower likelihood of access to basic chronic care in any country except Ghana, where people living in rural areas were significantly less likely to have access (Table 3).

**Table 2:** Health insurance, access to care and catastrophic expenditure for adults aged 50 years or older with chronic illness in six middle-income countries, 2007–2010

Indicator of universal health coverage	OR (95% CI)^a^ for indicator for insured^b^ versus uninsured participants^c^					
	China	Ghana	India	Mexico	Russian Federation^d^	South Africa
Access to basic chronic care^e^	1.54 (1.02 to 2.33)	1.69 (1.25 to 2.28)	3.03 (1.88 to 4.87)	2.73 (1.40 to 5.33)	N/A	1.01 (0.67 to 1.52)
Catastrophic health spending in last year^f^	1.50 (1.13 to 1.99)	1.22 (0.86 to 1.73)	1.96 (1.00 to 3.85)	0.49 (0.22 to 1.07)	N/A	3.39 (2.01 to 5.70)
Catastrophic out-of-pocket expenditure^g^	0.94 (0.54 to 1.63)	0.38 (0.23 to 0.62)	1.90 (1.14 to 3.17)	0.35 (0.14 to 0.84)	N/A	1.42 (0.38 to 5.25)

CI: confidence interval; N/A: not applicable; OR: odds ratio.

^a^ ORs and 95% CI were calculated using logistic regression models that controlled for sex, age, urban or rural residence, educational level, household income quintile and comorbidity.

^b^ Health insurance included voluntary and mandatory insurance.

^c^ Participants had reported being diagnosed with at least one chronic disease

^d^ As insurance coverage was almost universal in the Russian Federation no ORs could be calculated.

^e^ Basic chronic care included: (i) the provision of treatment, such as medications or advice on physical activity or diet, for each of the patient’s conditions; (ii) visiting outpatient services for the chronic condition or conditions one or more times in the last reported year; and (iii) maintenance of a stable health state after outpatient care.

^f^ Catastrophic health spending in the last year was defined as the household spending more on health in the last reported year than 30% of annual household income, after deduction of food expenditure.

^g^ Catastrophic out-of-pocket expenditure was defined as spending more on the last outpatient visit than 30% of annual household per capita income, after deduction of food expenditure.

**Table 3:** Area of residence and access to basic chronic care by adults aged 50 years or older with chronic illness in six middle-income countries, 2007–2010

Access to basic chronic care	China (*n* = 6558)	Ghana (*n* = 1327)	India (*n* = 2623)	Mexico (*n* = 1341)	Russian Federation (*n* = 2916)	South Africa (*n* = 1866)
Proportion of rural residents with access,^a^ % (95% CI)	30.3 (26.0 to 35.1)	30.0 (24.8 to 35.7)	30.4 (27.2 to 33.8)	31.5 (18.0 to 49.0)	46.6 (37.0 to 56.3)	52.1 (45.4 to 58.8)
Proportion of urban residents with access,^a^ % (95% CI)	30.7 (27.3 to 34.3)	43.2 (38.6 to 47.9)	38.0 (28.7 to 48.4)	18.1 (12.7 to 25.2)	42.2 (36.8 to 47.9)	45.5 (41.3 to 49.8)
Odds of access for rural versus urban residents, OR (95% CI)^b^	1.12 (0.85 to 1.48)	0.61 (0.45 to 0.84)	0.93 (0.60 to 1.45)	1.64 (0.83 to 3.28)	1.19 (0.78 to 1.81)	1.22 (0.86 to 1.75)

CI: confidence interval; OR: odds ratio.

^a^ Proportion of residents with access to basic chronic health care.

^b^ The ORs and 95% CIs were calculated using a logistic regression model that controlled for sex, age, health insurance, educational level, household income quintile and comorbidity.

Notes: Chronic illness was defined as being diagnosed with at least one chronic disease. Percentages are weighted.

Only 4.5% (95% CI: 3.0 to 6.7) of the participants in Ghana were dissatisfied or very dissatisfied with health-care services, as were only 6.3% (95% CI: 5.2 to 7.5) in China (Table 4, available at: http://www.who.int/bulletin/volumes/94/4/15-163832). The highest proportion who were dissatisfied or very dissatisfied was in Mexico (20.8%; 95% CI: 16.0 to 26.7), where, in addition, 19.1% (95% CI: 14.3 to 25.1) rated their involvement in health-care decision-making as “bad” or “very bad”. In China, Ghana, Mexico, the Russian Federation and South Africa, people who did not use outpatient care tended to be less satisfied with the health system than those who did (Table 4). 

**Table 4:** Dissatisfaction with the health system among adults aged 50 years or older with chronic illness in six middle-income countries, 2007–2010

Measure of dissatisfaction	China (*n* = 6558)	Ghana (*n* = 1327)	India (*n* = 2623)	Mexico (*n* = 1341)	Russian Federation (*n* = 2916)	South Africa (*n* = 1866)
**Dissatisfaction with health-care services^a^**						
Proportion dissatisfied, % (95% CI)	6.3 (5.2 to 7.5)	4.5 (3.0 to 6.7)	14.7 (12.0 to 17.8)	20.8 (16.0 to 26.7)	16.8 (12.5 to 22.3)	18.9 (15.6 to 22.6)
Risk of dissatisfaction for nonusers versus users of outpatient care,^b,c^ OR (95% CI)	1.56 (1.15 to 1.98)	1.61 (0.47 to 2.74)	1.05 (0.66 to 1.44)	1.65 (0.41 to 2.90)	1.68 (0.93 to 2.43)	1.39 (0.94 to 1.84)
**Insufficient involvement in health-care decision-making^d^**						
Proportion reporting insufficient involvement, % (95% CI)	5.0 (4.0 to 6.2)	9.8 (8.0 to 11.9)	13.7 (11.4 to 16.3)	19.1 (14.3 to 25.1)	15.5 (10.3 to 22.7)	19.7 (16.6 to 23.1)
Risk of insufficient involvement for nonusers versus users of outpatient care,^b,c^ OR (95% CI)	1.88 (1.24 to 2.53)	1.47 (0.83 to 2.11)	1.24 (0.77 to 1.72)	1.07 (0.37 to 1.77)	1.79 (0.87 to 2.72)	1.53 (1.10 to 1.96)

CI: confidence interval; OR: odds ratio.

^a^ An individual was regarded as being dissatisfied with health-care services if he or she reported being “dissatisfied” or “very dissatisfied” with the way health-care services were run.

^b^ ORs and 95% CI were calculated using a logistic regression model that controlled for household income quintile.

^c^ A user of outpatient care was defined as an individual who received outpatient care in the 12 months before the *Study on global ageing and adult health* survey.

^d^ An individual was regarded as having insufficient involvement in health-care decision-making if he or she rated their involvement in decisions about what services were provided and where they were provided as “bad” or “very bad”.

Note: Chronic illness was defined as being diagnosed with at least one chronic disease. Percentages are weighted.

**Fig. 7:**
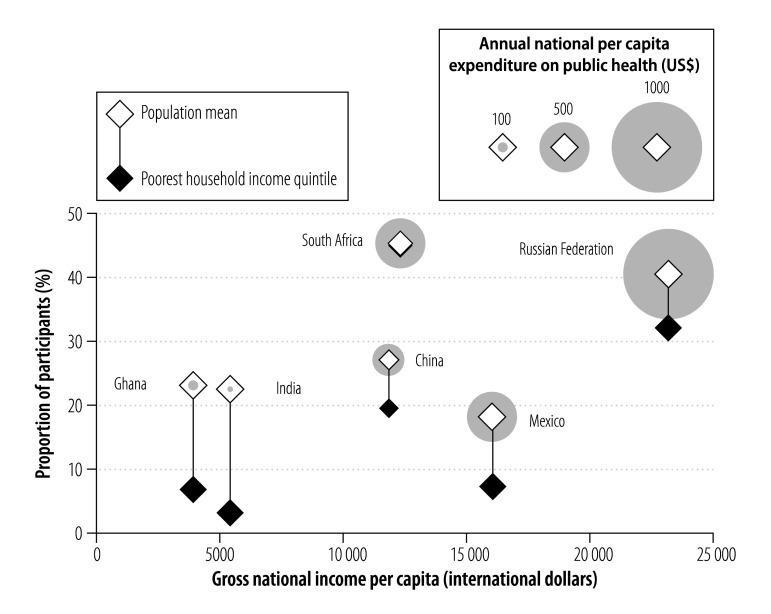
Access to basic chronic care without catastrophic expenditure for adults aged 50 years or older with chronic illness, by national income and health expenditure, in six middle-income countries, 2007–2010

The six countries in our study differed markedly in their level of economic and social development: for example, gross national income per capita in the Russian Federation was approximately six times that in Ghana (Table 5, available at: http://www.who.int/bulletin/volumes/94/4/15-163832). Generally, countries with larger socioeconomic inequalities had greater inequities in access to basic chronic care. The exception was South Africa, where access to basic chronic care was equally distributed across household income quintiles despite the country having one of the highest levels of social inequality in the world (Fig. 4). In fact, South Africa performed best in terms of achieving universal health coverage: 45.5% (95% CI: 41.1 to 50.0) of all participants had access to basic chronic care without incurring catastrophic out-of-pocket expenditure for the last outpatient visit (Fig. 7). The Russian Federation had the highest gross national income per capita and the highest public health expenditure per capita. Generally, access to basic chronic care without financial hardship in the country was similar to that in South Africa, but the poorest quintile was disadvantaged. Although China’s gross national income per capita was comparable to South Africa’s, both public health expenditure and the proportion of participants with access to basic chronic care without financial hardship were lower. Mexico had the second highest public health expenditure but performed poorly in terms of providing universal health coverage: only 7.3% (95% CI: 3.0 to 17.1) of the poorest quintile had access to basic chronic care without financial hardship and the proportion with access to basic chronic care without catastrophic out-of-pocket expenditure was the lowest of all six countries, including Ghana and India, which both had lower gross national incomes per capita.

**Table 5:** National macroeconomic and social indicators in six middle-income countries, 2007–2010

National indicator	China	Ghana	India	Mexico	Russian Federation	South Africa
**Macroeconomic**						
Gross national income per capita in 2013, international dollars^a^	11 850	3900	5350	16 110	23 190	12 240
Public health expenditure as a fraction of gross domestic product in 2012, (%)	3.0	3.0	1.3	3.2	3.8	4.2
Public health expenditure per capita in 2012, (US$)	331.8	110.4	68.8	515.3	896.7	515.9
Out-of-pocket payments as a fraction of total health expenditure in 2012, (%)	34.3	28.7	57.6	44.1	34.3	7.2
**Social**						
Gini coefficient^b^ (year estimated)	0.37 (2011)	0.43 (2006)	0.34 (2012)	0.48 (2012)	0.40 (2009)	0.65 (2011)
Income share held by richest 10% of population (year estimated)	30.0 (2010)	32.8 (2006)	28.8 (2010)	38.9 (2012)	31.0 (2009)	53.8 (2011)

US$: United States dollars.

^a^ Figures were adjusted for purchasing power parity.

^b^ The Gini coefficient measures the extent to which the distribution of income or consumption expenditure among individuals or households within an economy deviates from a perfectly equal distribution: a coefficient of 0 represents perfect equality, whereas a coefficient of 1 implies perfect inequality.

Data source: World Bank.^11^^–^^17^

US$: United States dollars.

Notes: Chronic illness was defined as being diagnosed with at least one chronic disease. Catastrophic out-of-pocket expenditure was defined as expenditure more than 30% of annual household per capita income, after the deduction of food expenditure at the last outpatient visit. Household income quintiles were based on annual household per capita income. Figures for gross national income per capita were adjusted for purchasing power parity.

## Discussion

The prevalence of diagnosed chronic conditions in people aged 50 years or more varied widely among the six study countries and was higher in the more developed and more urbanized areas. This may be due to the negative health impact of the lifestyle changes accompanying modernization.^18^ None of the six countries provided access to basic chronic care for more than half the participants, which is in line with evidence of gaps in essential services for noncommunicable diseases in low- and middle-income countries.^19^ Although it is often assumed that wealthier countries may be better at providing health services for noncommunicable diseases, we found no evidence that a higher level of development was associated with greater universal health coverage.^20^

The inequities in health coverage we observed in middle-income countries and that have been reported in other studies persist despite substantial health reforms aimed at improving universal health coverage, especially for poor and vulnerable groups.^21^^–^^24^ The poor chronically ill were less likely to receive basic chronic care and more likely to face financial hardship than the better off in all countries in our study, except in South Africa, where primary health care is provided free to all citizens.^25^ Although previous evidence suggests that rural residents have more limited access to primary care than urban residents and are less likely to have health insurance,^26^ we found that participants in rural and urban areas had similar access to basic chronic care in all study countries except Ghana. More detailed country-specific data are required to explore this potential difference in greater depth.

In Mexico, we found that almost 80% of the participants did not have access to basic chronic care, which is consistent with the Mexican President Peña Nieto’s statement in 2013 that much of the population cannot exercise their right to health.^27^ In 2014, the Mexican National Council for the Evaluation of Social Development Policy reported that only 21.4% of the population received medical care for their health problems but that between 84% and 97% of people with health problems did receive medical treatment.^28^ The inconsistency may be due to the difference between perceived and medically defined health needs. Since individuals’ experiences and expectations of the probable outcome of medical care can shape perceptions of their health status, many older people may not think their health problem requires medical care despite being diagnosed with a chronic condition.^29^ We found that people who were dissatisfied with the health system were less likely to seek care despite medical need. The same appears to be true for people who perceive it as ineffective.^30^ In India, the common belief that the private sector offers better quality care coupled with inadequate public provision has led many people to use private facilities and bear high out-of-pocket costs despite increased investment in public health and the exemption of vulnerable groups from user fees.^4^

We found that both the insured and uninsured could experience financial hardship in all study countries. Although health insurance improved access to health care, it also increased the risk of catastrophic health spending in most countries. In the Russian Federation, universal health insurance became mandatory in 1993 and health services are provided free at the point of care. However, the cost of pharmaceuticals excluded from guaranteed packages and informal payments can result in catastrophic expenditure.^23^ In Ghana, where a national health insurance scheme was established in 2003, we found that insurance increased access to basic chronic care and protected against catastrophic out-of-pocket expenditure for the last outpatient visit, in agreement with the previous findings.^31^ However, insured households were more likely to incur catastrophic spending during the last year, perhaps due to more frequent service utilization by the insured.^31^

In China, health insurance did not significantly influence the likelihood of catastrophic out-of-pocket expenditure for the last outpatient visit but the insured were more likely to experience catastrophic spending during the last year, as reported previously.^32^^,^^33^ This suggests that recent social health insurance programmes in China have neither reduced the risk of catastrophic spending nor relieved the financial burden on older people with chronic conditions. In India and South Africa, a small minority of insured people had an increased risk of catastrophic spending – they were mainly covered by private insurance schemes that may have encouraged the use of specialist providers with higher co-payments.^21^^,^^34^ In Mexico, insurance generally increased protection against financial hardship. However, our findings provide only partial evidence that the voluntary *Seguro Popular* insurance scheme introduced in 2003 protected against financial hardship because health service utilization was extremely low, especially among the poor, and little information was available on insurance schemes in Mexico’s highly fragmented health insurance system.^35^^,^^36^

Our study had several limitations. The WHO Study on Global Ageing and Adult Health provides the best, available, comparable data on older adults with chronic illness in middle-income countries because it uses a unified method but the self-reported prevalence of noncommunicable disease is less than the actual prevalence in older people. In particular, in some countries the poor are less likely to be given a diagnosis.^20^ Accordingly, the level of access to basic chronic care may have been overestimated and, consequently, inequities may have been underestimated. Although the national representativeness of the household survey could have been weakened by the variation in response rate between countries, the results of our sensitivity analysis confirmed the validity of the samples (data available from the corresponding author).

Universal health coverage remains a distant hope for many older adults with chronic illness in middle-income countries. Although allocating a higher share of a country’s gross national income to health might improve services and subsidize health care for the poor, economic development does not in itself guarantee universal health coverage or greater equity. Nevertheless, lower socioeconomic inequality generally leads to more equal distribution of health services. Yet, as evident in South Africa, the provision of free primary health care can help achieve equitable universal health coverage despite high socioeconomic inequality, which suggests that universal social protection may guard better against catastrophic expenditure than insurance schemes. If gaps in universal health coverage are to be closed, it is essential that the care provided is acceptable to the population. Consequently, health reforms should aim to improve service quality and promote democratic oversight of health care through increased social participation in addition to expanding insurance schemes. The provision of universal health coverage for older people with chronic conditions is particularly challenging for low- and middle-income countries, especially given the ongoing epidemiological transition. It is crucial, therefore, that future health policies are tailored to the specific needs of older people.
